# Effect of timing, technique and molecular features on brain control with local therapies in oncogene-driven lung cancer

**DOI:** 10.1016/j.esmoop.2021.100161

**Published:** 2021-06-02

**Authors:** R.A. El Shafie, K. Seidensaal, F. Bozorgmehr, D. Kazdal, T. Eichkorn, M. Elshiaty, D. Weber, M. Allgäuer, L. König, K. Lang, T. Forster, N. Arians, S. Rieken, C.-P. Heussel, F.J. Herth, M. Thomas, A. Stenzinger, J. Debus, P. Christopoulos

**Affiliations:** 1 Department of Radiation Oncology, Heidelberg University Hospital, Heidelberg, Germany; 2 National Center for Radiation Oncology (NCRO), Heidelberg Institute for Radiation Oncology (HIRO), Heidelberg, Germany; 3 Department of Radiology and Nuclear Medicines, Thoraxklinik at Heidelberg University Hospital, Heidelberg, Germany; 4 Department of Thoracic Oncology, Thoraxklinik at Heidelberg University Hospital, Heidelberg, Germany; 5 Translational Lung Research Center Heidelberg (TLRC-H), Member of the German Center for Lung Research (DZL), Heidelberg, Germany; 6 Institute of Pathology, Heidelberg University Hospital, Heidelberg, Germany; 7 Institute of Medical Biometry and Informatics (IMBI), Heidelberg University Hospital, Heidelberg, Germany; 8 University Medical Center Göttingen, Department of Radiation Oncology, Göttingen, Germany; 9 Department of Pneumology, Thoraxklinik at Heidelberg University Hospital, Heidelberg, Germany; 10 National Center for Tumor Diseases (NCT), Heidelberg, Germany; 11 Clinical Cooperation Unit Radiation Oncology (E050), German Cancer Research Center (DKFZ), Heidelberg, Germany; 12 Deutsches Konsortium für Translationale Krebsforschung (DKTK), Partner Site Heidelberg, German Cancer Research Center (DKFZ), Heidelberg, Germany; 13 Heidelberger Ionenstrahltherapie-Zentrum (HIT), Heidelberg, Germany

**Keywords:** EGFR^+^ NSCLC, ALK^+^ NSCLC, brain metastases, whole-brain radiotherapy, stereotactic radiotherapy

## Abstract

**Background:**

The improved efficacy of tyrosine kinase inhibitors (TKI) mandates reappraisal of local therapy (LT) for brain metastases (BM) of oncogene-driven non-small-cell lung cancer (NSCLC).

**Patients and methods:**

This study included all epidermal growth factor receptor-mutated (EGFR^+^, *n* = 108) and anaplastic lymphoma kinase-rearranged (ALK^+^, *n* = 33) TKI-naive NSCLC patients diagnosed with BM in the Thoraxklinik Heidelberg between 2009 and 2019. Eighty-seven patients (62%) received early LT, while 54 (38%) received delayed (*n* = 34; 24%) or no LT (*n* = 20; 14%). LT comprised stereotactic (SRT; *n* = 40; 34%) or whole-brain radiotherapy (WBRT; *n* = 77; 66%), while neurosurgical resection was carried out in 19 cases.

**Results:**

Median overall survival (OS) was 49.1 months for ALK^+^ and 19.5 months for EGFR^+^ patients (*P* = 0.001), with similar median intracranial progression-free survival (icPFS) (15.7 versus 14.0 months, respectively; *P* = 0.80). Despite the larger and more symptomatic BM (*P* < 0.001) of patients undergoing early LT, these experienced longer icPFS [hazard ratio (HR) 0.52; *P* = 0.024], but not OS (HR 1.63; *P* = 0.12), regardless of the radiotherapy technique (SRT versus WBRT) and number of lesions. High-risk oncogene variants, i.e. non-del19 *EGFR* mutations and ‘short’ *EML4-ALK* fusions (mainly variant 3, E6:A20), were associated with earlier intracranial progression (HR 2.97; *P* = 0.001). The longer icPFS with early LT was also evident in separate analyses of the EGFR^+^ and ALK^+^ subsets.

**Conclusions:**

Despite preferential use for cases with poor prognostic factors, early LT prolongs the icPFS, but not OS, in TKI-treated EGFR^+^/ALK^+^ NSCLC. Considering the lack of survival benefit, and the neurocognitive effects of WBRT, patients presenting with polytopic BM may benefit from delaying radiotherapy, or from radiosurgery of multiple or selected lesions. For SRT candidates, the improved tumor control with earlier radiotherapy should be weighed against the potential toxicity and the enhanced intracranial activity of newer TKI. High-risk *EGFR*/*ALK* variants are associated with earlier intracranial failure and identify patients who could benefit from more aggressive management.

## Highlights

• Upfront LT delay intracranial progression, but do not prolong OS in oncogene-driven NSCLC.
• Use of stereotactic versus whole-brain radiotherapy and primary tumor *TP53* status do not significantly affect brain control.
• Non-del19 *EGFR* mutations and ‘short’ *EML4-ALK* fusions are independent predictors of earlier intracranial failure.

## Introduction

Approximately 20% of non-small-cell lung cancers (NSCLC) harbor actionable driver mutations (dm-NSCLC), with important consequences for patient management and outcome.^1^ Most frequent are mutations in the epidermal growth factor receptor gene (*EGFR*) occurring in 10%-15% of adenocarcinomas, followed by anaplastic lymphoma kinase gene (*ALK*) fusions in up to 5%.^2^ Approximately 25% of these patients present with brain metastases (BM) at the time of diagnosis, which appear, at least in EGFR^+^ tumors, to be associated with a worse prognosis.^3^, ^4^, ^5^ The strategies to treat central nervous system (CNS) involvement in dm-NSCLC are influenced by the increasing availability of targeted drugs with better CNS penetration and antitumor activity than conventional chemotherapy.^5^, ^6^, ^7^ In particular, the next-generation tyrosine kinase inhibitors (TKI) osimertinib, alectinib, brigatinib, and lorlatinib achieve durable CNS responses in ~80% of patients compared with 40%-60% for older targeted substances.^8^, ^9^, ^10^, ^11^ Besides, median overall survival (OS) currently exceeds 3 years for EGFR^+^ and 5 years for ALK^+^ NSCLC patients,^12^^,^^13^ which increases the risk of long-term toxicity from local CNS treatments (LT).^14^^,^^15^ Therefore, the timing and technique of LT for BM in dm-NSCLC are highly controversial. Conflicting results have been reported in previous retrospective analyses in EGFR^+^ NSCLC regarding the ability of LT to improve intracranial tumor control and OS,^16^, ^17^, ^18^, ^19^ while no similar study exists for ALK^+^ patients, and prospective data are lacking. Moreover, even though molecular tumor properties are increasingly recognized as crucial determinants of clinical outcome in both EGFR^+^ and ALK^+^ NSCLC,^4^^,^^20^, ^21^, ^22^ their potential importance for management of brain disease remains unclear. The current study addresses these questions by analyzing consecutive patients treated over 10 years in a large European thoracic oncology center.

## Patients and methods

### Study population, treatment, and data collection

This study was approved by the Heidelberg University ethics committee (S-172/2018 and S-145/2017) and carried out following institutional guidelines and the Declaration of Helsinki in its current version. Since this was a non-interventional, retrospective study, informed consent was obtained whenever possible, but was not required for every participant.

Included were all EGFR^+^/ALK^+^ NSCLC patients diagnosed with BM at our institution between 2009 and 2019. Patient characteristics and treatment details were systematically collected from the medical records.^23^ Histological diagnosis and molecular profiling of NSCLC were carried out at the Institute of Pathology, Heidelberg University Hospital, according to the criteria of the current WHO Classification (2015) for lung cancer.^24^ Next-generation sequencing (NGS) was carried out on a semiconductor-based platform (ThermoFisher Scientific, Waltham, MA) with custom panels covering 38-42 genes considered relevant for lung cancer biology, which included all *EGFR* exons, and *TP53* exons 4-10 (DNA-based), as well as known *ALK* fusion variants (RNA-based), as published.^25^ The decision for early (i.e. within 30 days of TKI start) versus delayed LT was made by our Multidisciplinary Thoracic Oncology Tumor Board (MTB) considering various parameters, for example, the presence or absence of symptoms (with preferential use of early LT for symptomatic patients), or the location of brain lesions (with preferential use of early LT for infratentorial lesions). The decision for stereotactic radiotherapy (SRT) versus whole-brain radiotherapy (WBRT) was also made by our MTB, which recommended SRT for ≤4 metastases until 2016 (in accordance with the guidelines of the German Society for Radio-Oncology),^26^ and for ≤10 metastases thereafter. For SRT, either stereotactic radiosurgery (SRS) or hypofractionated stereotactic radiotherapy (HFSRT), the CyberKnife M6 system or a linear accelerator (LINAC) adapted for radiosurgery were used. Target volume delineation and treatment planning for SRT were based on high-resolution contrast-enhanced computed tomography (CT), as well as magnetic resonance imaging (MRI) scans and carried out as previously described.^27^^,^^28^ Employed safety margins depended on treatment technique and ranged between 1 mm for CyberKnife and 2-3 mm for LINAC-based radiosurgery. The target volume for post-operative SRT encompassed the resection cavity with a safety margin of 3-4 mm. Post-operative cavities were treated with HFSRT, as were lesions with a diameter larger than 3 cm. SRT doses ranged between 18 and 20 Gy margin dose with prescription to the enclosing 70% isodose for CyberKnife treatments and 80% isodose for LINAC-based radiosurgery. Doses for HFSRT ranged between 30 and 35 Gy in 6-7 fractions. WBRT was delivered using conventional techniques at typically 30 Gy in 10 fractions, five times a week. Alternate dose regimens of 35 Gy in 14 fractions or 40-42 Gy in 20-21 fractions were used in eight cases. Follow-up consisted of regular high-resolution cranial MRI and/or contrast-enhanced CT scans. Intracranial tumor status was assessed by neuro-radiologists according to the criteria for Response Assessment in Neuro-Oncology for brain metastases (RANO-BM).^29^

### Statistical analysis

Descriptive statistics for baseline variables included mean (with standard deviation, SD) and/or median values (with range or interquartile range, IQR) for continuous variables, and absolute or relative frequencies for categorical variables. Follow-up time was calculated using the reverse Kaplan–Meier (KM) method.^30^ OS was calculated from BM diagnosis to the date of death or last follow-up. Intracranial progression-free survival (icPFS) was calculated from BM diagnosis to last imaging follow-up or radiologic progression. OS and icPFS were analyzed according to KM. The prognostic influence of baseline characteristics on OS and icPFS was analyzed using proportional hazards Cox regression. Baseline variables were systematically examined for their relationship with clinical endpoints in order to uncover potential confounders. Multivariable modeling included parameters showing significant associations with outcome in univariable analysis, and those of special clinical interest. Variable selection was verified with a component-wise gradient boosting algorithm to optimize the C-index^31^^,^^32^ using the R-package mboost with a step length of 0.0002 and initial number of iterations of 2000 for OS and 1000 for icPFS, due to different numbers of patients and events. Since this is a retrospective exploratory data analysis, *P* values are of descriptive nature. Statistical analyses were carried out with the R software (v.3.6.2; R Core Team, 2019) and SPSS v24 (IBM, Armonk, NY)*.*

## Results

### Patient characteristics

Between 2009 and 2019, 179 EGFR^+^/ALK^+^ NSCLC patients were diagnosed with BM at our institution, of which 79% (*n* = 141) were TKI-naive and included in this study. Patient characteristics are detailed in Table 1. One hundred and eight patients (77%) had *EGFR*-mutated tumors, mostly with exon19 deletions (*n* = 61; 57%), while 33 patients (23%) featured *ALK* rearrangements. Fifty-four patients (38%) presented with ≥5 BM. For 87 patients (62%) LT was given early, i.e. upon detection of BM, whereas for 54 (38%) LT was given delayed, i.e. upon subsequent progression under systemic treatment (*n* = 34; 24%), or not at all (*n* = 20; 14%). LT consisted of stereotactic radiotherapy (SRT; *n* = 40; 34%) or WBRT (*n* = 77; 66%). Neurosurgical resection was carried out with (*n* = 15; 11%) or without (*n* = 4; 3%) post-operative radiotherapy (PORT) in 19 cases. TKI treatment comprised first (*n* = 93; 66%), second (*n* = 31; 22%), or third generation compounds (*n* = 17; 12%; details given in the footnote of Table 1). Decision for early versus delayed LT was significantly associated with presence of symptomatic (51% versus 12%, *P* < 0.001) and larger BM (average maximum diameter 18.3 versus 9.7 mm, *P* < 0.001), neurosurgical resection (18.4% versus 5.6%, *P* = 0.03), and administration of WBRT (66.7% versus 35.2%, *P* < 0.001, Table 1). In addition, there was a trend for more frequent use of steroids before radiotherapy (RT) (51% versus 31%, *P* = 0.164) and more frequent polytopic disease (i.e. ≥5 lesions, 41% versus 33%, *P* = 0.34) in patients with early LT (Table 1).

**Table 1:** Baseline characteristics

	Delayed LT (*n* = 54)	Early LT (*n* = 87)	Total (*n* = 141)	*P* value
Age at BM diagnosis, years				
Median (Q1-Q3)	59 (54-69)	60 (51-68)	60 (52-68)	0.652
Sex				
Female, *n* (%)	35 (64.8)	60 (69.0)	95 (67.4)	0.609
Mutation				
*ALK n* (%)	15 (27)	18 (20.7)	33 (23.4)	0.334
*TP53* mutated^a^	3/15	4/18		0.876
*EGFR n* (%)	39 (72.2)	69 (79.3)	108 (76.6)	
*TP53* mutated^a^	15/39	26/69		0.936
High-risk oncogene variant				
Short *EML4-ALK* (*n* = 13)^b^	5/13	8/13		0.239
Non-del19 *EGFR*^mut^ (*n* = 47)^c^	19/47	28/47		0.063
ECOG performance status (missing)	(1)	(3)	(4)	
0 *n* (%)	33 (62.3)	43 (51.2)	76 (55.5)	0.204
≥1 *n* (%)	20 (37.7)	41 (48.8)	61 (44.5)	
Stage at initial diagnosis				
I-III *n* (%)	9 (16.7)	8 (9.2)	17 (12.1)	0.185
IV *n* (%)	45 (83.3)	79 (90.8)	124 (87.9)	
SCS simplified comorbidity score (missing)	(16)	(18)	(34)	
Mean (SD) *n* (%)	4.2 (3.8)	3.8 (3.6)	3.9 (3.7)	0.656
Min-max	0.0-13.0	0.0-10.0	0.0-13.0	
Surgery				
Primary tumor *n* (%)	5 (9.3)	10 (11.5)	15 (10.6)	0.676
BM (neurosurgery) *n* (%)	3 (5.6)	16 (18.4)	19 (13.5)	0.030
Timepoint of BM diagnosis				
Synchronous *n* (%)	13 (24.1)	11 (12.6)	24 (17.0)	0.079
Metachronous *n* (%)	41 (75.9)	76 (87.4)	117 (83.0)	
Number of BM				
Solitary (*n* = 45)	18/45	27/45	87 (61.7)	0.056
1-4 *n* (%)	36 (66.7)	51 (58.6)	87 (61.7)	0.339
≥5 *n* (%)	18 (33.3)	36 (41.4)	54 (38.3)	
Maximum size (diameter) of BM in mm				
Mean (SD)	9.7 (7.0)	18.3 (12.3)	15.0 (11.4)	<0.001
Symptomatic BM (missing)	(3)	(3)	(6)	
Yes *n* (%)	6 (11.8)	43 (51.2)	49 (36.3)	<0.001
No *n* (%)	45 (88.2)	41 (48.8)	86 (63.7)	
Steroid treatment before RT (missing)	(19)	(9)	(28)	
*n* (%)	13 (37)	40 (51)	53 (47)	0.164
Radiotherapy technique				
SRS *n* (%)	15 (27.8)	25 (29.0)	40 (28.4)	0.968
WBRT *n* (%)	19 (35.2)	58 (66.7)	77 (54.6)	<0.001
None *n* (%)	20 (37.0)	4 (4.6)	24 (17.0)	<0.001
TKI generation^d^				
First *n* (%)	32 (59.3)	61 (70.1)	93 (66.0)	0.163
Second *n* (%)	12 (22.2)	19 (21.8)	31 (22.0)	
Third *n* (%)	10 (18.5)	7 (8.0)	17 (12.1)	

Statistical comparison between the ‘early’ and ‘delayed’ subgroups were carried out with the chi-square test for categorical and *t*-test for continuous variables.

ALK, anaplastic lymphoma kinase; BM, brain metastases; del19, exon 19 deletion; ECOG, Eastern Cooperative Oncology Group; EGFR, epidermal growth factor receptor; RT, radiotherapy; SD, standard deviation; SRS, stereotactic radiosurgery; TKI, tyrosine kinase inhibitor; WBRT, whole-brain radiotherapy.

a *TP53* status at diagnosis available for 107 patients (48/107 mutated, 7/28 ALK^+^ and 41/79 EGFR^+^).

b High-risk *EML4-ALK*: 12x E6:A20 (V3), and 1x E9:A20 (short fusions); data available for 24/33 (also 8x V1, and 3x V2).

c High-risk *EGFR* variants: all non-del19 mutations (47/108).

d ALK: 28/33 crizotinib, 4/33 ceritinib, 1/33 alectinib; EGFR: 65/108 erlotinib/gefitinib, 28/108 afatinib, 15/108 osimertinib.

### OS

**Figure 1:**
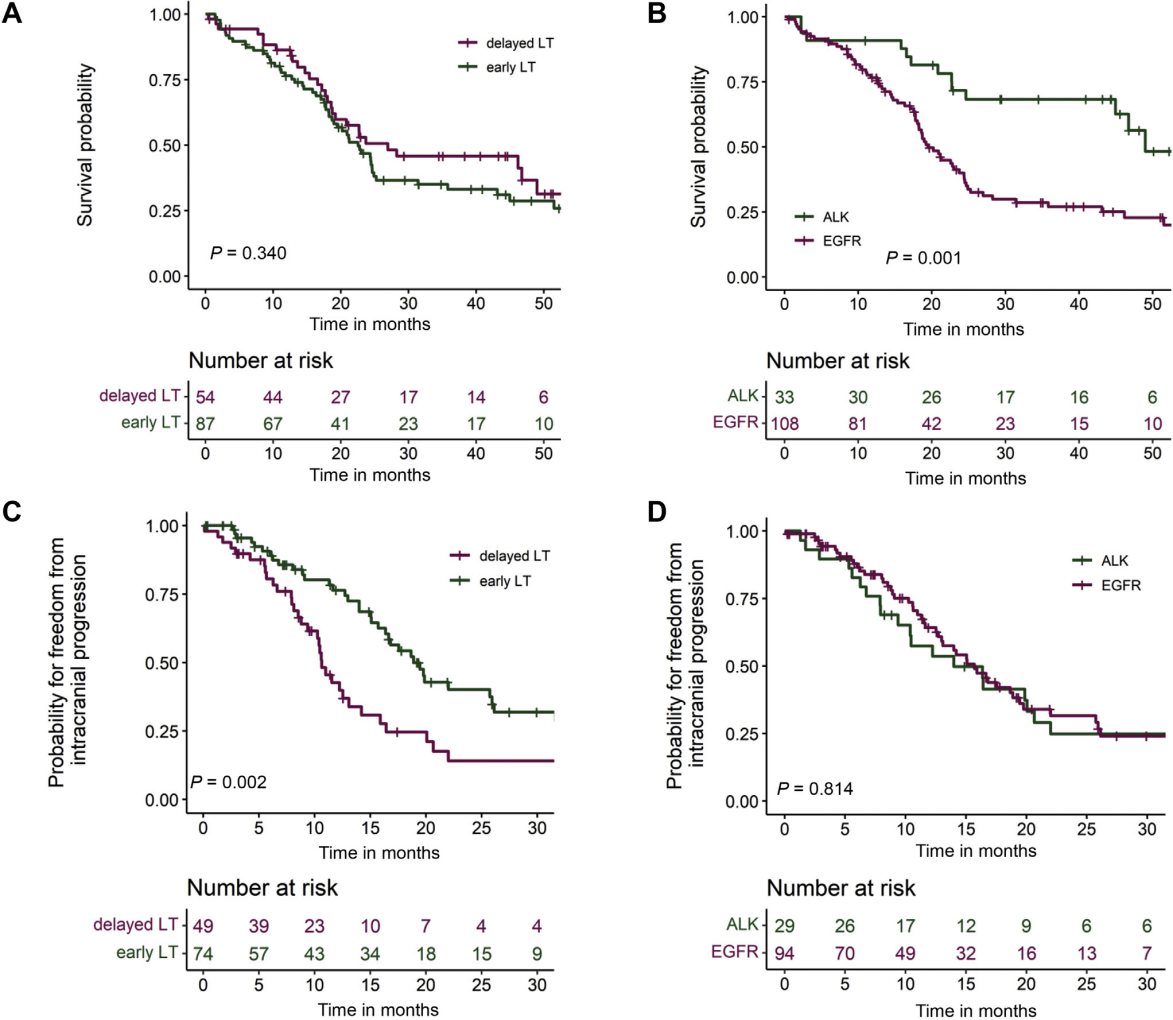
Overall and intracranial progression-free survival according to the timing of local therapy and oncogenic driver in non-small-cell lung cancer. (A) Median overall survival (OS) was 22.6 months [95% confidence interval (CI) 18.5-26.7 months] for patients with early local therapy (LT) versus 27.0 months (95% CI 4.4-49.9 months) for patients with delayed LT (*P* = 0.340, Table 2). (B) Median OS was 19.5 months (95% CI 17.0-22.0 months) for epidermal growth factor receptor gene mutated (EGFR)^+^ patients versus 49.0 months (95% CI 38.2-59.8 months) for anaplastic lymphoma kinase rearranged (ALK)^+^ patients (*P* = 0.001, Table 2). (C) Median intracranial progression-free survival (icPFS) was 19.4 months (95% CI 16.3-22.6 months) for patients with early LT versus 10.6 months (95% CI 9.4-11.8 months) patients with delayed LT (*P* = 0.002, Table 2). (D) Median icPFS was 15.7 months (95% CI 12.8-18.7 months) for EGFR^+^ patients versus 14.0 months (95% CI 7.1-20.9 months) for ALK^+^ patients (*P* = 0.814, Table 2).

Median follow-up time for OS was 44.3 months (IQR: 29.3-61.3) for the entire cohort. At the time of analysis, 85 patients had died and 56 patients were still alive, corresponding to an OS of 80.2% at 12 months [KM estimate; 95% confidence interval (CI): 73.7-87.2], 48.3% at 24 months (KM estimate; 95% CI: 40.2-58.1) and a median OS of 23.0 months (IQR: 14.5-54.4). Median OS did not significantly differ between the early-LT and delayed-LT subgroups, with 22.6 months (IQR: 13.2-56.3) versus 27.0 months (IQR: 16.6-54.4), respectively [Figure 1A; Table 2: hazard ratio (HR) 1.24; 95% CI: 0.79-2.0; *P* = 0.340]. In multivariable analysis, ALK^+^ showed a longer median OS compared to EGFR^+^ patients (Figure 1B: 49.0 versus 19.5 months; Table 2: HR 2.34; 95% CI: 1.10-5.22; *P* = 0.028), while neurosurgical BM resection was also favorable, (HR 0.26; 95% CI: 0.10-0.70; *P* = 0.007, Table 2). Presence of *TP53* mutations was significantly associated with shorter OS (HR 1.85; 95% CI: 1.05-3.25; *P* = 0.033), while the adverse effect of unfavorable oncogene variants, i.e. *EGFR* mutations other than exon 19 deletions (non-del19) and ‘short’ *EML4-ALK* fusions, mainly variant 3 (V3, E6:A20), on OS was less pronounced and evident as a trend (HR 1.66; *P* = 0.081, Table 2). Neither the application of WBRT versus SRT, nor the number of BM, maximum BM diameter, presence of symptoms, or use of steroids before RT were significantly associated with OS (Table 2).

**Table 2:** Overall and intracranial progression-free survival in the entire study population

Entire study population	Univariable analysis			Multivariable analysis^a^		
Overall survival	HR	95% CI	*P* value	HR	95% CI	*P* value
Sex (male versus female)	0.94	(0.59-1.50)	0.792	—	—	—
Mutation (EGFR versus ALK)	2.61	(1.50-4.70)	0.001	2.34	(1.10-5.22)	0.028
High-risk variant (non-del19/V3)	1.47	(0.95-2.27)	0.084	1.66	(0.94-2.93)	0.081
*TP53* mutated at diagnosis	1.81	(1.01-3.08)	0.026	1.85	(1.05-3.25)	0.033
ECOG performance status ≥1	1.53	(0.99-2.40)	0.054	1.15	(0.65-2.04)	0.622
Stage IV at initial diagnosis	1.26	(0.63-2.50)	0.519	—	—	—
Simplified comorbidity score (SCS)	1.01	(0.95-1.10)	0.752	—	—	—
Synchronous BM diagnosis	0.88	(0.52-1.50)	0.639	—	—	—
Multiple BM (≥5)	1.11	(0.71-1.70)	0.651	—	—	—
Solitary BM	0.90	(0.57-1.40)	0.637	—	—	—
Maximum BM size (≥12 versus <12 mm)^b^	0.94	(0.60-1.47)	0.788	—	—	—
Symptomatic BM	1.13	(0.72-1.80)	0.590	—	—	—
Steroid treatment before RT	1.34	(0.84-2.12)	0.221	—	—	—
SRS versus no RT	0.59	(0.27-1.26)	0.173	—	—	—
WBRT versus no RT	1.20	(0.74-1.94)	0.460	—	—	—
WBRT versus SRT	0.77	(0.48-1.25)	0.293	—	—	—
Second versus first-generation TKI	0.67	(0.39-1.16)	0.154	—	—	—
Third versus first-generation TKI	0.70	(0.25-1.95)	0.496	—	—	—
Neurosurgical resection	0.36	(0.16-0.78)	0.009	0.26	(0.10-0.70)	0.007
Early LT (versus delayed)	1.24	(0.79-2.00)	0.340	1.63	(0.89-3.00)	0.117

Intracranial PFS	HR	95% CI	*P* value	HR	95% CI	*P* value
Sex (male versus female)	1.18	(0.73-1.90)	0.501	—	—	—
Mutation (*EGFR* versus *ALK*)	1.06	(0.64-1.80)	0.814	—	—	—
High-risk variant (non-del19/V3)	2.53	(1.53-4.18)	<0.001	2.96	(1.60-5.47)	0.001
*TP53* mutated at diagnosis	1.46	(0.85-2.51)	0.166	1.36	(0.77-2.39)	0.290
ECOG performance status ≥1	0.94	(0.59-1.50)	0.803	—	—	—
Stage IV at initial diagnosis	0.85	(0.39-1.90)	0.679	—	—	—
Surgery carried out on primary tumor	0.50	(0.22-1.20)	0.108	—	—	—
Synchronous BM diagnosis	0.64	(0.37-1.10)	0.122	—	—	—
Multiple BM (≥5 versus 1-4)	1.31	(0.81-2.10)	0.273	—	—	—
Solitary BM	0.52	(0.31-0.87)	0.013	0.37	(0.19-0.72)	0.004
Symptomatic BM	0.70	(0.42-1.20)	0.173	—	—	—
SRS versus no RT	0.53	(0.24-1.19)	0.123	—	—	—
WBRT versus no RT	0.78	(0.47-1.29)	0.331	—	—	—
WBRT versus SRT	1.31	(0.80-2.15)	0.290	—	—	—
Second versus first-generation TKI	0.85	(0.50-1.43)	0.529	—	—	—
Third versus first-generation TKI	0.31	(0.08-1.27)	0.103	—	—	—
Highly CNS-active TKI^c^	0.73	(0.43-1.24)	0.244	0.56	(0.31-1.03)	0.062
Neurosurgical resection	0.60	(0.31-1.10)	0.117	—	—	—
Early LT (versus delayed)	0.47	(0.30-0.76)	0.002	0.52	(0.30-0.92)	0.024

ALK, anaplastic lymphoma kinase; BM, brain metastases; CI, confidence interval; CNS, central nervous system; del19, exon 19 deletion; ECOG, Eastern Cooperative Oncology Group; EGFR, epidermal growth factor receptor; HR, hazard ratio; LT, local therapy; PFS, progression-free survival; RT, radiotherapy; SRS, stereotactic radiosurgery; SRT, stereotactic radiotherapy; TKI, tyrosine kinase inhibitor; WBRT, whole-brain radiotherapy.

a Multivariable analysis included timing of local treatment (early versus delayed LT), technique of radiotherapy (WBRT versus SRT versus no RT), as well as parameters with statistical significance in univariable testing, or of special clinical importance.

b Dichotomized at the median value of 12 mm.

c Osimertinib, or any ALK inhibitor other than crizotinib.

### Intracranial progression-free survival

IcPFS was 62.5% at 12 months (95% CI 53.6% to 72.8%), 29.3% at 24 months (95% CI 20.8% to 41.2%), and 15.7 months in median for the entire cohort (IQR: 8.9-26.1 months). In multivariable analysis, early LT was significantly associated with longer icPFS compared with delayed LT (Figure 1C: 10.6 versus 18.9 months; Table 2: HR 0.52; 95% CI 0.30-0.92; *P* = 0.024), but the technique of radiotherapy (WBRT versus SRT) had no significant influence (HR 0.77; 95% CI 0.48-1.25; *P* = 0.293). No relevant difference in icPFS was detected between the EGFR^+^ and ALK^+^ subgroups (Figure 1D: HR 1.06; 95% CI 0.64-1.80; *P* = 0.814). In addition, presence of a solitary BM was favorable (HR 0.37 versus presence of multiple BM; 95% CI 0.19-0.72; *P* = 0.004), while presence of high-risk oncogene variants (HR 2.96; 95% CI 1.60-5.47; *P* = 0.001), but not of *TP53* mutations (*P* = 0.290), was associated with earlier intracranial progression (Table 2).

### Subgroup analyses for EGFR^+^ and ALK^+^ patients

**Figure 2:**
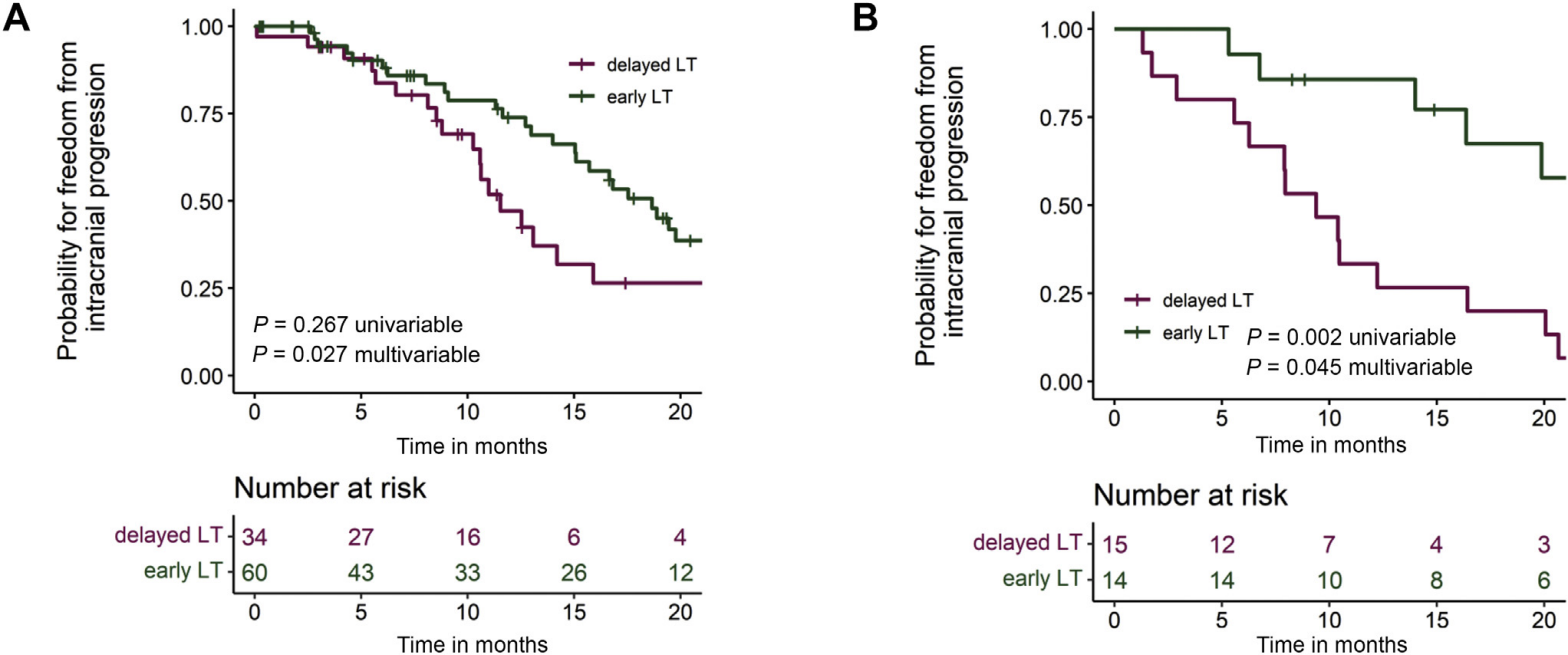
Intracranial progression-free survival for epidermal growth factor receptor gene mutated (EGFR)^+^ and anaplastic lymphoma kinase rearranged (ALK)^+^ lung cancer patients according to the timing of local therapy. (A) Median intracranial progression-free survival (icPFS) was 18.7 months [95% confidence interval (CI) 15.8-21.6 months] for EGFR^+^ patients with early local therapy (LT) versus 11.5 months (95% CI 8.8-14.2 months) for EGFR^+^ patients with delayed LT (*P* = 0.267 in univariable testing, and *P* = 0.027 in multivariable testing, Table 3). (B) Median icPFS was 36.0 months (CI not available due to the low number of events) for ALK^+^ patients with early LT versus 9.4 months (95% CI 5.5-13.2 months) for ALK^+^ patients with delayed LT (*P* = 0.002 in univariable and *P* = 0.045 in multivariable testing, Table 3).

Separate subgroup analyses of icPFS were carried out for EGFR^+^ and ALK^+^ patients (Figure 2, Table 3), including the timing (early versus late) and technique (WBRT versus SRT) of LT, as well as parameters significantly linked to icPFS in the entire cohort, i.e. oncogene variant, and presence of a solitary BM (Table 2). Similar to the findings in the entire cohort, early LT was significantly associated with a longer icPFS in both the EGFR^+^ (HR 0.50; 95% CI 0.26-0.96; *P* = 0.038) and ALK^+^ patient subsets (HR 0.27; 95% CI 0.76-0.97; *P* = 0.045), while there were no differences according to the employed RT technique (WBRT versus SRT). In addition, the oncogene variant showed significant associations with icPFS in univariable analysis for both the EGFR^+^ (HR 2.40; 95% CI 1.33-4.35; *P* = 0.004) and ALK^+^ subsets (HR 2.87; 95% CI 1.02-8.05; *P* = 0.045) (Supplementary Figure S1, available at https://doi.org/10.1016/j.esmoop.2021.100161), but in multivariable testing remained significant only for EGFR^+^ patients (HR 3.05; 95% CI 1.55-6.00; *P* = 0.001), presumably due the small size of the ALK^+^ cohort (Table 3). Similar to the results in the entire cohort, early LT did not affect OS within the EGFR^+^ and ALK^+^ subsets (Supplementary Figure S2, available at https://doi.org/10.1016/j.esmoop.2021.100161).

**Table 3:** Subgroup analyses for intracranial progression-free survival in EGFR^+^/ALK^+^ patients

Intracranial progression-free survival^a^	Univariable analysis			Multivariable analysis		
EGFR^+^ NSCLC subgroup	HR	95% CI	*P* value	HR	95% CI	*P* value
Early LT (versus delayed)	0.72	0.40-1.29	0.267	0.50	0.26-0.96	0.038
WBRT versus SRT	1.46	0.80-2.67	0.221	—	—	—
High-risk variant (non-del19)	2.40	1.33-4.35	0.004	3.05	1.55-6.00	0.001
Solitary BM	0.46	0.24-0.87	0.017	0.56	0.28-1.13	0.107

ALK^+^ NSCLC subgroup	HR	95% CI	*P* value	HR	95% CI	*P* value
Early LT (versus delayed)	0.20	0.07-0.56	0.002	0.27	0.76-0.97	0.045
WBRT versus SRT	0.95	0.37-2.42	0.909	—	—	—
High-risk *ALK* variant	2.87	1.02-8.05	0.045	1.73	0.56-5.37	0.342
Solitary BM	0.62	0.25-1.56	0.309	—	—	—

ALK, anaplastic lymphoma kinase; BM, brain metastases; CI, confidence interval; EGFR, epidermal growth factor receptor; HR, hazard ratio; LT, local therapy; NSCLC, non-small-cell lung cancer; SRT, stereotactic radiotherapy; TKI, tyrosine kinase inhibitor; WBRT, whole-brain radiotherapy.

a Multivariable analysis included timing of local treatment (early versus delayed LT), technique of radiotherapy (WBRT versus SRT), as well as parameters with statistical significance in the entire study population (Table 2).

## Discussion

The optimal timing and technique of LT in dm-NSCLC with BM are subjects of ongoing debate. Our study shows that early LT improves icPFS, but not OS of EGFR^+^ and ALK^+^ patients, independent of the RT technique, i.e. WBRT versus SRT (Tables 2 and 3). Therefore, the previously postulated special effect of WBRT on icPFS due to the eradication of diffuse micrometastases^33^^,^^34^ might be less relevant in dm-NSCLC patients, in which the superior brain efficacy of TKI compared with chemotherapy additionally contributes to intracranial tumor control. Along the same lines, the presence of ≤4 versus >4 brain lesions did not influence OS or icPFS in our study either, supporting the notion that the degree of initial intracranial spread is not crucial.^35^ An important exception was patients with a solitary BM, who have been highlighted as a particularly favorable subgroup in previous reports and had longer icPFS in our study, as well (Table 2).^36^ Considering the lack of efficacy advantage for WBRT over SRT, and its deleterious effect on cognitive function and quality of life,^14^^,^^15^^,^^37^^,^^38^ WBRT should be avoided whenever possible, and delayed LT might be reasonable when application of SRT is technically limited due to a very large number of lesions. Of note, while use of SRT was restricted to patients with 1-3 metastases in the past, it is currently being extended with equivalent OS results for up to 10 BM, and its role for >10 BM is also being reappraised.^35^^,^^39^, ^40^, ^41^ Emerging individualized concepts for extensive, polytopic brain involvement include SRT for the symptomatic, largest or fastest progressing lesions, in analogy to the concept of oligoprogression,^42^ and control of the remaining BM by CNS-active systemic therapies. However, the exact workflows and patient selection criteria for application remain to be defined, ideally in the form of prospective clinical trials. When WBRT is inevitable, hippocampal avoidance in combination with memantine can also reduce cognitive impairment.^43^^,^^44^ It is worth noting that the addition of EGFR TKI to WBRT does not further increase neurotoxicity according to a systematic review.^45^

Another important issue is whether upfront LT could be altogether omitted in favor of TKI-only treatment. Prospective evidence to support this strategy is available only for asymptomatic BM from EGFR^+^/ALK^+^ NSCLC, and only with first-line use of the newer TKI osimertinib, alectinib, brigatinib, or lorlatinib, which show very good intracranial efficacy.^8^, ^9^, ^10^, ^11^ For symptomatic BM, surgery and/or radiotherapy are generally preferable in order to quickly reduce mass effects to the healthy brain and alleviate neurological symptoms.^6^ Also, for EGFR^+^ patients with BM receiving first-/second-generation EGFR inhibitors, a meta-analysis of 1086 patients from seven studies showed that upfront RT in addition to TKI resulted in a better icPFS and OS compared with TKI only, especially if the number of BMs was limited.^16^ Similar results were also observed in a second meta-analysis of 24 studies including 2810 EGFR^+^ patients: RT plus first-/second-generation TKI resulted in a better response, longer OS, and longer icPFS than TKI monotherapy.^46^ However, other investigators have reported different results, for example, that the OS benefit from a combined upfront RT/TKI approach is restricted to patients with 1-4 BM only according to a retrospective analysis of 176 EGFR^+^ cases,^17^ or that upfront WBRT/TKI does not improve OS, but only icPFS, and this only for patients with >3 BM.^19^ In our study, early LT improved icPFS, but not OS in EGFR^+^ and ALK^+^ NSCLC with BM, regardless of the number of lesions (≤4 versus >4) and the applied technique (WBRT versus SRT, Tables 2 and 3). Summarizing the available evidence, prolongation of icPFS from a combined upfront RT/TKI treatment of BM in dm-NSCLC appears to be reproducible, but the potential OS benefit remains controversial and becomes less likely, as more potent EGFR (osimertinib) and ALK inhibitors (alectinib, brigatinib, lorlatinib) enter the first-line setting. Prospective data are urgently needed to clarify the exact conditions under which upfront RT might be safely omitted in favor of TKI-only treatment with these newer compounds, as well as the neurocognitive side-effects and influence on quality of life for each strategy. Such a randomized phase II study of EGFR^+^ patients is currently ongoing (OUTRUN, NCT03497767).

For ALK^+^ NSCLC, data on the effect of early versus delayed LT on patient survival are scarce. Despite the lack of OS benefit, early brain radiotherapy prolonged icPFS in our study and should therefore be considered for crizotinib-treated patients, which comprised the majority (*n* = 28/33; 85%) of our ALK^+^ cases.^47^^,^^48^ The improved intracranial control when adding RT to crizotinib was also evident in a retrospective analysis of patients enrolled in the PROFILE 1005 and 1007 studies.^49^ At the mechanistic level, the tumoricidal effect of radiotherapy not only complements the weak activity of crizotinib, which achieves a brain response rate of only 30%-50% as monotherapy,^49^^,^^50^ but also increases permeability of the blood-brain barrier, as could be shown in pharmacokinetic studies.^51^ On the other hand, newer ALK inhibitors show intracranial response rates comparable to these of radiotherapy, i.e. ~80% (44), which could obviate benefit from additional RT, as suggested by a small retrospective series of patients treated with brain-penetrant TKI presented recently.^52^ Real-world data are not yet mature for this comparison, because the time since first-line approval of alectinib and brigatinib (December 2018 and May 2020, respectively in Europe) is still shorter than their median first-line PFS (>2 years). Whether cranial RT can safely be deferred for newly diagnosed ALK^+^ NSCLC patients with BM receiving highly brain-active TKI, remains unclear and will need to be addressed in future studies.

To our knowledge, our study is the first to highlight the impact of molecular tumor characteristics on intracranial disease control. While non-del19 *EGFR* mutations, ‘short’ *EML4-ALK* fusions (mainly variant 3), and presence of *TP53* co-mutations have all been linked to earlier systemic treatment failure in both EGFR^+^ and ALK^+^ NSCLC,^53^, ^54^, ^55^, ^56^ our results show that high-risk oncogene variants are more important that *TP53* status for intracranial disease control (Table 2 and Supplementary Figure S1, available at https://doi.org/10.1016/j.esmoop.2021.100161). This is in accordance with the lack of association between *TP53* mutations and brain involvement in both EGFR^+^ and ALK^+^ NSCLC, while unfavorable oncogene variants increase metastatic potential (*EML4-ALK* V3) and/or decrease TKI sensitivity (*EML4-ALK* V3 and non-del19 *EGFR* mutations).^57^, ^58^, ^59^ Therefore, patients with these molecular alterations constitute a higher ‘brain risk’ population, which would probably benefit more from closer radiologic monitoring as well as earlier and more aggressive LT.

The main limitations of our study are its retrospective character and relatively small number of patients, especially with the rare ALK^+^ disease. Given the heterogeneity of our cohort (Table 1), potential confounders were controlled by inclusion in multivariable modeling (Table 2). For the interpretation of our results, it is important to consider that the decisions for WBRT versus SRT, as well as for early versus delayed LT are frequently linked to the presence of unfavorable BM characteristics,^60^^,^^61^ such as larger and/or polytopic lesions, more symptomatic and/or requiring steroids, which was also evident in our cohort (Table 1). Despite this poor prognostic profile of BM, early LT could improve intracranial disease control, and thus also prevent earlier BM-related death, which highlights the exquisite antitumor potency of radiotherapy. Nonetheless, the fact that this prolongation of icPFS did not translate to a longer OS (Table 2) suggests that adverse biologic characteristics of tumors causing aggressive brain involvement and necessitating early LT, presumably facilitate progression at other (extracranial) sites, which then becomes the limiting factor for survival. The main strengths of our study are the homogeneous management of our patients, who were consecutive, TKI-naive, and treated at the same large tertiary cancer center; the standardized molecular profiling with combined DNA/RNA NGS also carried out for all patients in the same institution; the systematic in-house MRI-based follow-up; as well as a dedicated clinical registry, which ensured consistency of data capture and processing.^23^ Furthermore, the present study is to our knowledge the first real-world analysis to include a reasonably sized ALK^+^ NSCLC cohort evaluable for OS, and the first to systematically examine the effect of molecular tumor characteristics on intracranial disease control.

In summary, a combined TKI/early LT strategy in EGFR^+^ and ALK^+^ NSCLC with BM improves icPFS but not OS, regardless of the radiotherapy technique (SRT or WBRT) and number of brain lesions. This could be linked to the generally more adverse prognostic profile of patients chosen for the early LT strategy in the clinical routine. Considering the lack of OS benefit and the toxicities of WBRT compared with SRT, decisions about the timing and technique of radiotherapy in dm-NSCLC should be individualized based on the patient's life expectancy. In the particular case of polytopic BM early-on, WBRT should be avoided by delaying RT under MRI surveillance or by SRT of multiple BM. High-risk oncogene variants, i.e. non-del19 *EGFR* mutations and *EML4-ALK* V3, confer earlier intracranial failure and identify patients who could benefit from more aggressive surveillance and treatment strategies.

## References

[1] Rosell R., Karachaliou N. (2016). Large-scale screening for somatic mutations in lung cancer. Lancet.

[2] Griesinger F., Eberhardt W., Nusch A. (2020). Biomarker testing in non-small cell lung cancer in routine care: analysis of the first 3,717 patients in the German prospective, observational, nation-wide CRISP Registry (AIO-TRK-0315). Lung Cancer.

[3] Doebele R.C., Lu X., Sumey C. (2012). Oncogene status predicts patterns of metastatic spread in treatment-naive nonsmall cell lung cancer. Cancer.

[4] Christopoulos P., Kirchner M., Roeper J. (2020). Risk stratification of EGFR+ lung cancer diagnosed with panel-based next-generation sequencing. Lung Cancer.

[5] Preusser M., Winkler F., Valiente M. (2018). Recent advances in the biology and treatment of brain metastases of non-small cell lung cancer: summary of a multidisciplinary roundtable discussion. ESMO Open.

[6] Nabors L.B., Portnow J., Ahluwalia M. (2020). Central nervous system cancers, Version 3.2020, NCCN clinical practice guidelines in oncology. J Natl Compr Canc Netw.

[7] Berghoff A.S., Preusser M. (2018). New developments in brain metastases. Ther Advanc Neurol Disord.

[8] Reungwetwattana T., Nakagawa K., Cho B.C. (2018). CNS response to osimertinib versus standard epidermal growth factor receptor tyrosine kinase inhibitors in patients with untreated EGFR-mutated advanced non-small-cell lung cancer. J Clin Oncol.

[9] Peters S., Camidge D.R., Shaw A.T. (2017). Alectinib versus crizotinib in untreated ALK-positive non-small-cell lung cancer. N Engl J Med.

[10] Shaw A.T., Bauer T.M., Marinis F.D. (2020). First-line lorlatinib or crizotinib in advanced ALK-positive lung cancer. N Engl J Med.

[11] Camidge D.R., Kim H.R., Ahn M.J. (2018). Brigatinib versus crizotinib in ALK-positive non-small-cell lung cancer. N Engl J Med.

[12] Ramalingam S.S., Vansteenkiste J., Planchard D. (2020). Overall survival with osimertinib in untreated, EGFR-mutated advanced NSCLC. N Engl J Med.

[13] Mok T., Camidge D.R., Gadgeel S.M. (2020). Updated overall survival and final progression-free survival data for patients with treatment-naive advanced ALK-positive non-small-cell lung cancer in the ALEX study. Ann Oncol.

[14] Chang E.L., Wefel J.S., Hess K.R. (2009). Neurocognition in patients with brain metastases treated with radiosurgery or radiosurgery plus whole-brain irradiation: a randomised controlled trial. Lancet Oncol.

[15] Brown P.D., Jaeckle K., Ballman K.V. (2016). Effect of radiosurgery alone vs radiosurgery with whole brain radiation therapy on cognitive function in patients with 1 to 3 brain metastases: a randomized clinical trial. J Am Med Assoc.

[16] Wang C., Lu X., Lyu Z. (2018). Comparison of up-front radiotherapy and TKI with TKI alone for NSCLC with brain metastases and EGFR mutation: a meta-analysis. Lung Cancer.

[17] Miyawaki E., Kenmotsu H., Mori K. (2019). Optimal sequence of local and EGFR-TKI therapy for EGFR-mutant non-small cell lung cancer with brain metastases stratified by number of brain metastases. Int J Radiat Oncol Biol Phys.

[18] Magnuson W.J., Lester-Coll N.H., Wu A.J. (2017). Management of brain metastases in tyrosine kinase inhibitor-naïve epidermal growth factor receptor-mutant non-small-cell lung cancer: a retrospective multi-institutional analysis. J Clin Oncol.

[19] He Z.-Y., Li M.-F., Lin J.-H. (2019). Comparing the efficacy of concurrent EGFR-TKI and whole-brain radiotherapy vs EGFR-TKI alone as a first-line therapy for advanced EGFR-mutated non-small-cell lung cancer with brain metastases: a retrospective cohort study. Cancer Manag Res.

[20] Passaro A., Malapelle U., Del Re M. (2020). Understanding EGFR heterogeneity in lung cancer. ESMO Open.

[21] Camidge D.R., Niu H., Kim H.R. (2020). Correlation of baseline molecular and clinical variables with ALK inhibitor efficacy in ALTA-1L. J Clin Oncol.

[22] Christopoulos P., Budczies J., Kirchner M. (2019). Defining molecular risk in ALK(+) NSCLC. Oncotarget.

[23] Kessel K.A., Bohn C., Engelmann U. (2014). Five-year experience with setup and implementation of an integrated database system for clinical documentation and research. Comput Methods Programs Biomed.

[24] Travis W.D., Brambilla E., Nicholson A.G. (2015). The 2015 World Health Organization classification of lung tumors: impact of genetic, clinical and radiologic advances since the 2004 classification. J Thor Oncol.

[25] Volckmar A.L., Leichsenring J., Kirchner M. (2019). Combined targeted DNA and RNA sequencing of advanced NSCLC in routine molecular diagnostics: analysis of the first 3,000 Heidelberg cases. Int J Cancer.

[26] Kocher M., Wittig A., Piroth M.D. (2014). Stereotactic radiosurgery for treatment of brain metastases: a report of the DEGRO Working Group on Stereotactic Radiotherapy. Strahlenther Onkol.

[27] El Shafie R.A., Tonndorf-Martini E., Schmitt D. (2019). Pre-operative versus post-operative radiosurgery of brain metastases-volumetric and dosimetric impact of treatment sequence and margin concept. Cancers.

[28] El Shafie R.A., Paul A., Bernhardt D. (2018). Evaluation of stereotactic radiotherapy of the resection cavity after surgery of brain metastases compared to postoperative whole-brain radiotherapy (ESTRON)-a single-center prospective randomized trial. Neurosurgery.

[29] Lin N.U., Lee E.Q., Aoyama H. (2015). Response assessment criteria for brain metastases: proposal from the RANO group. Lancet Oncol.

[30] Schemper M., Smith T.L. (1996). A note on quantifying follow-up in studies of failure time. Control Clin Trials.

[31] Mayr A., Hofner B., Schmid M. (2016). Boosting the discriminatory power of sparse survival models via optimization of the concordance index and stability selection. BMC Bioinformatics.

[32] Hofner B., Mayr A., Robinzonov N. (2014). Model-based boosting in R: a hands-on tutorial using the R package mboost. Comput Stat.

[33] Kocher M., Soffietti R., Abacioglu U. (2011). Adjuvant whole-brain radiotherapy versus observation after radiosurgery or surgical resection of one to three cerebral metastases: results of the EORTC 22952-26001 study. J Clin Oncol.

[34] Mahajan A., Ahmed S., McAleer M.F. (2017). Post-operative stereotactic radiosurgery versus observation for completely resected brain metastases: a single-centre, randomised, controlled, phase 3 trial. Lancet Oncol.

[35] Yamamoto M., Serizawa T., Shuto T. (2014). Stereotactic radiosurgery for patients with multiple brain metastases (JLGK0901): a multi-institutional prospective observational study. Lancet Oncol.

[36] Qin H., Wang C., Jiang Y. (2015). Patients with single brain metastasis from non-small cell lung cancer equally benefit from stereotactic radiosurgery and surgery: a systematic review. Med Sci Monit.

[37] Aoyama H., Tago M., Kato N. (2007). Neurocognitive function of patients with brain metastasis who received either whole brain radiotherapy plus stereotactic radiosurgery or radiosurgery alone. Int J Radiat Oncol Biol Phys.

[38] Brown P.D., Ballman K.V., Cerhan J.H. (2017). Postoperative stereotactic radiosurgery compared with whole brain radiotherapy for resected metastatic brain disease (NCCTG N107C/CEC·3): a multicentre, randomised, controlled, phase 3 trial. Lancet Oncol.

[39] El Shafie R.A., Celik A., Weber D. (2020). A matched-pair analysis comparing stereotactic radiosurgery with whole-brain radiotherapy for patients with multiple brain metastases. J Neurooncol.

[40] Robin T.P., Camidge D.R., Stuhr K. (2018). Excellent outcomes with radiosurgery for multiple brain metastases in ALK and EGFR driven non-small cell lung cancer. J Thor Oncol.

[41] El Shafie R.A., Paul A., Bernhardt D. (2019). Robotic radiosurgery for brain metastases diagnosed with either SPACE or MPRAGE sequence (CYBER-SPACE)-a single-center prospective randomized trial. Neurosurgery.

[42] Guckenberger M., Lievens Y., Bouma A.B. (2020). Characterisation and classification of oligometastatic disease: a European Society for Radiotherapy and Oncology and European Organisation for Research and Treatment of Cancer consensus recommendation. Lancet Oncol.

[43] Brown P.D., Gondi V., Pugh S. (2020). Hippocampal avoidance during whole-brain radiotherapy plus memantine for patients with brain metastases: phase III trial NRG oncology CC001. J Clin Oncol.

[44] Grosu A.-L., Frings L., Bentsalo I. (2020). Whole-brain irradiation with hippocampal sparing and dose escalation on metastases: neurocognitive testing and biological imaging (HIPPORAD) – a phase II prospective randomized multicenter trial (NOA-14, ARO 2015-3, DKTK-ROG). BMC Cancer.

[45] Hendriks L.E.L., Schoenmaekers J., Zindler J.D. (2015). Safety of cranial radiotherapy concurrent with tyrosine kinase inhibitors in non-small cell lung cancer patients: a systematic review. Cancer Treat Rev.

[46] Wang X., Xu Y., Tang W. (2018). Efficacy and safety of radiotherapy plus EGFR-TKIs in NSCLC patients with brain metastases: a meta-analysis of published data. Transl Oncol.

[47] Wang W., Sun X., Hui Z. (2019). Treatment optimization for brain metastasis from anaplastic lymphoma kinase rearrangement non-small-cell lung cancer. Oncol Res Treat.

[48] McCusker M.G., Russo A., Scilla K.A. (2019). How I treat ALK-positive non-small cell lung cancer. ESMO Open.

[49] Costa D.B., Shaw A.T., Ou S.-H.I. (2015). Clinical experience with crizotinib in patients with advanced ALK-rearranged non-small-cell lung cancer and brain metastases. J Clin Oncol.

[50] Elsayed M., Christopoulos P. (2021). Therapeutic sequencing in ALK+ NSCLC. Pharmaceuticals.

[51] Metro G., Lunardi G., Floridi P. (2015). CSF concentration of crizotinib in two ALK-positive non-small-cell lung cancer patients with CNS metastases deriving clinical benefit from treatment. J Thorac Oncol.

[52] Thomas N.J., Myall N.J., Sun F. (2020). Time to first progression in patients with NSCLC with brain metastases receiving next generation TKI alone vs TKI + brain radiation. WCLC.

[53] Canale M., Petracci E., Delmonte A. (2017). Impact of TP53 mutations on outcome in EGFR-mutated patients treated with first-line tyrosine kinase inhibitors. Clin Cancer Res.

[54] Christopoulos P., Kirchner M., Bozorgmehr F. (2019). Identification of a highly lethal V3^+^TP53^+^ subset in ALK^+^ lung adenocarcinoma. Int J Cancer.

[55] Woo C.G., Seo S., Kim S.W. (2017). Differential protein stability and clinical responses of EML4-ALK fusion variants to various ALK inhibitors in advanced ALK-rearranged non-small cell lung cancer. Ann Oncol.

[56] Christopoulos P., Kirchner M., Endris V. (2018). EML4-ALK V3, treatment resistance, and survival: refining the diagnosis of ALK+ NSCLC. J Thorac Dis.

[57] Christopoulos P., Endris V., Bozorgmehr F. (2018). *EML4-ALK* fusion variant V3 is a high-risk feature conferring accelerated metastatic spread, early treatment failure and worse overall survival in ALK^+^ NSCLC. Int J Cancer.

[58] O'Regan L., Barone G., Adib R. (2020). EML4-ALK V3 oncogenic fusion proteins promote microtubule stabilization and accelerated migration through NEK9 and NEK7. J Cell Sci.

[59] Kobayashi Y., Mitsudomi T. (2016). Not all epidermal growth factor receptor mutations in lung cancer are created equal: perspectives for individualized treatment strategy. Cancer Sci.

[60] Sperduto P.W., Kased N., Roberge D. (2012). Summary report on the graded prognostic assessment: an accurate and facile diagnosis-specific tool to estimate survival for patients with brain metastases. J Clin Oncol.

[61] Steindl A., Yadavalli S., Gruber K.-A. (2020). Neurological symptom burden impacts survival prognosis in patients with newly diagnosed non-small cell lung cancer brain metastases. Cancer.

