# Supplementary material for: Effect of timing, technique and molecular features on brain control with local therapies in oncogene-driven lung cancer
Source: ESMO Open. 2021 Jun 2;6(3):100161. doi: 10.1016/j.esmoop.2021.100161 (PMC8182387; doi:10.1016/j.esmoop.2021.100161)

Supplementary Material

Supplementary Figure 1. Intracranial progression-free survival of EGFR+ and ALK+ patients according to oncogene variant. (A) Median intracranial progression-free survival (icPFS) was 18.9 months (95% confidence interval [CI] 12.3-25.5) for patients with *EGFR* exon 19 deletions (del19) *vs.* 11.6 months (CI 8.6-14.6) for patients with other *EGFR* mutations (p=0.004, Table 3) (B) Median icPFS was 10.4 months (CI 7.5-13.2) for patients with "short" *EML4-ALK* variants (mainly V3, Table 1) *vs.* 20.1 months (CI 14.5-26.6) for patients with other *EML4-ALK* variants (p=0.045, Table 3).

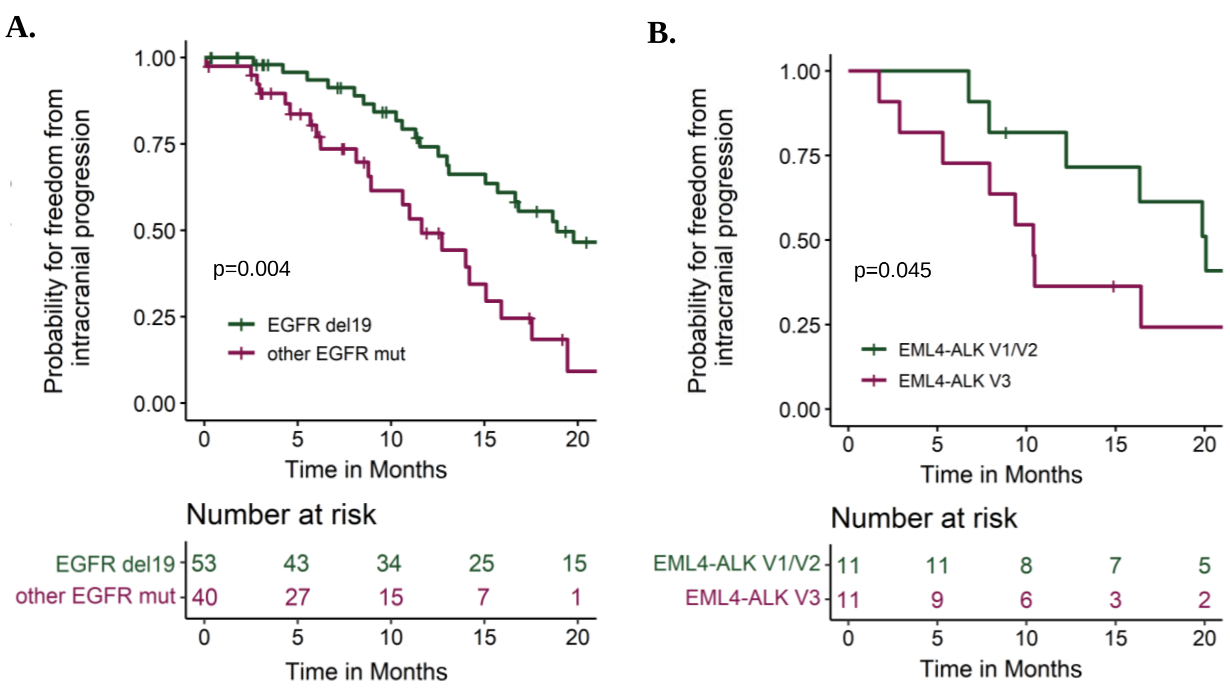

Supplementary Figure 2. Overall survival of EGFR^{+} and ALK^{+} patients with brain metastases according to the timing of local therapy. (A) Median overall survival (OS) was 20.2 months (95% confidence interval [CI] 17.3-23.1) for EGFR^{+} patients with early local therapy (LT) *vs.* 19.2 months (CI 11.9-26.5) for EGFR^{+} patients with delayed LT (p=0.415) (B) Median OS was 56.3 months (CI 35.7-76.8) for ALK^{+} patients with early LT *vs.* 49.0 months (CI 19.5-78.4) for ALK^{+} patients with delayed LT (p=0.789).

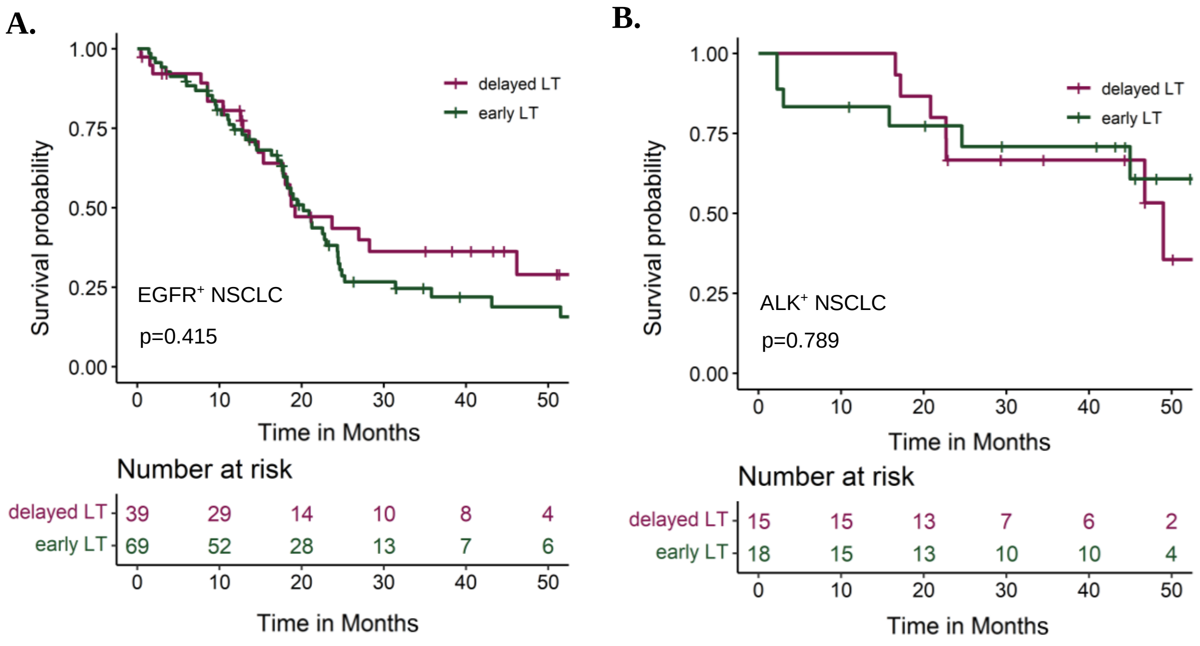

Supplement: Supplementary Material [file mmc1.pdf]
